# Supplementary figures and images for: Beta2 Oscillations in Hippocampal-Cortical Circuits During Novelty Detection
Source: Front Syst Neurosci. 2021 Feb 16;15:617388. doi: 10.3389/fnsys.2021.617388 (PMC7921172; doi:10.3389/fnsys.2021.617388)

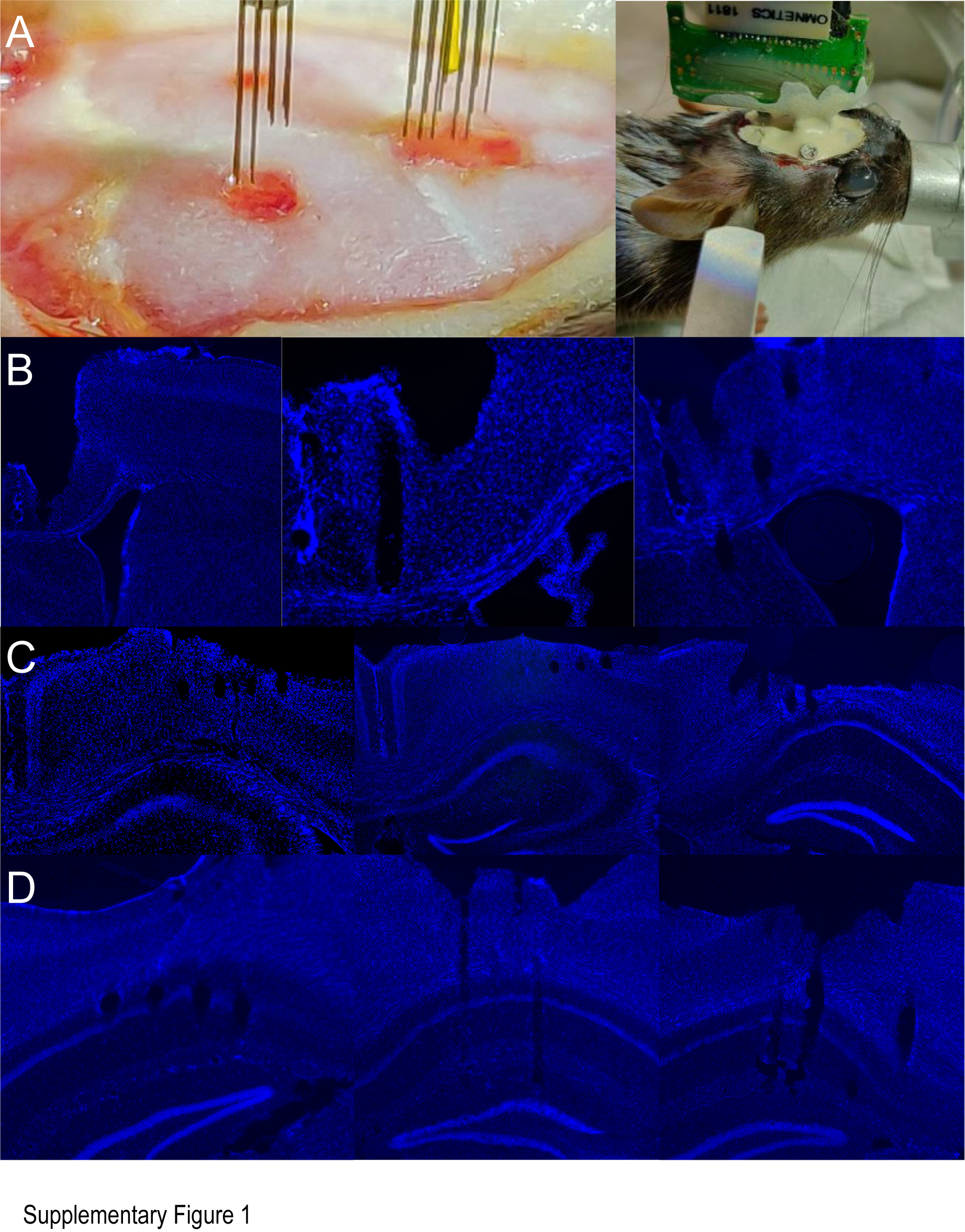

Supplement: Supplementary file 1 [file Image_1.TIF]

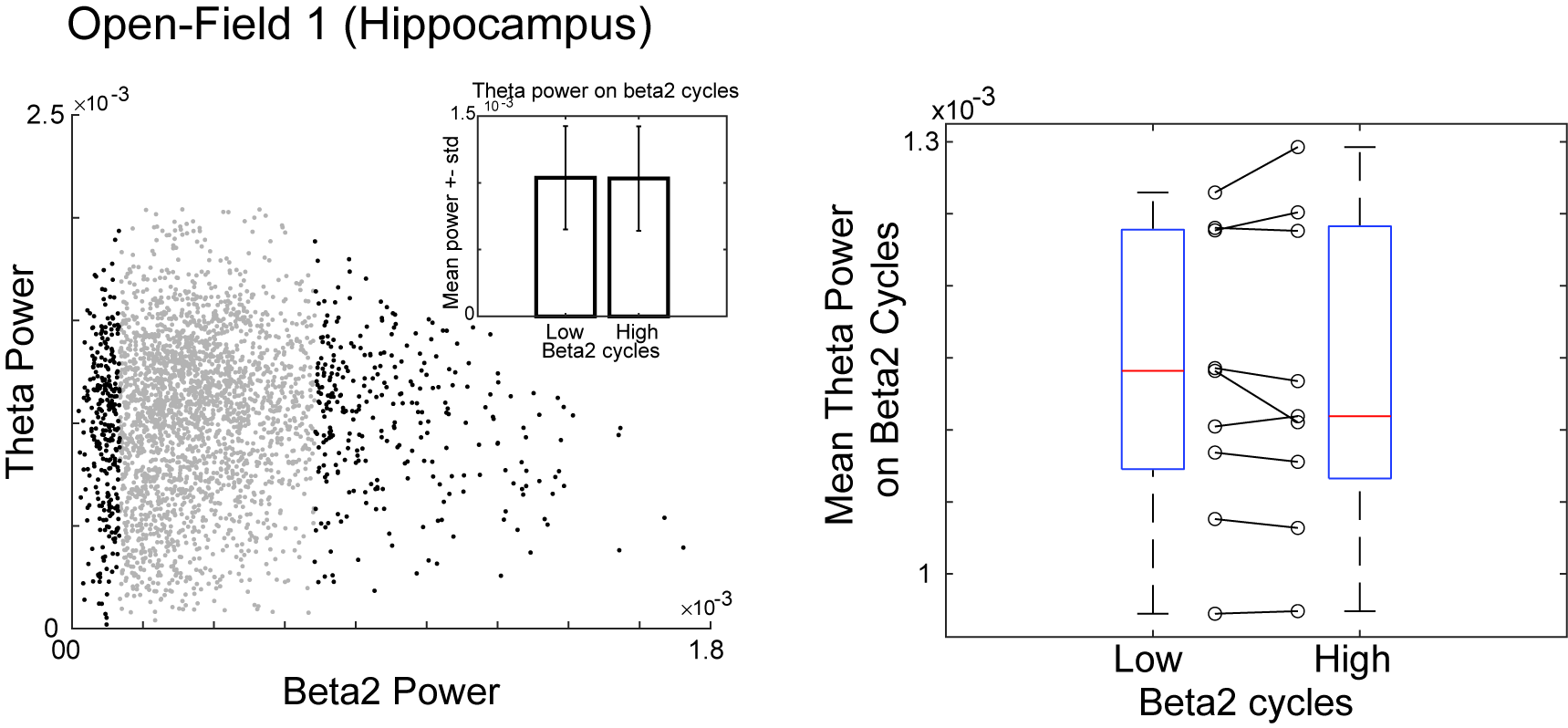

Supplement: Supplementary file 2 [file Image_2.TIF]

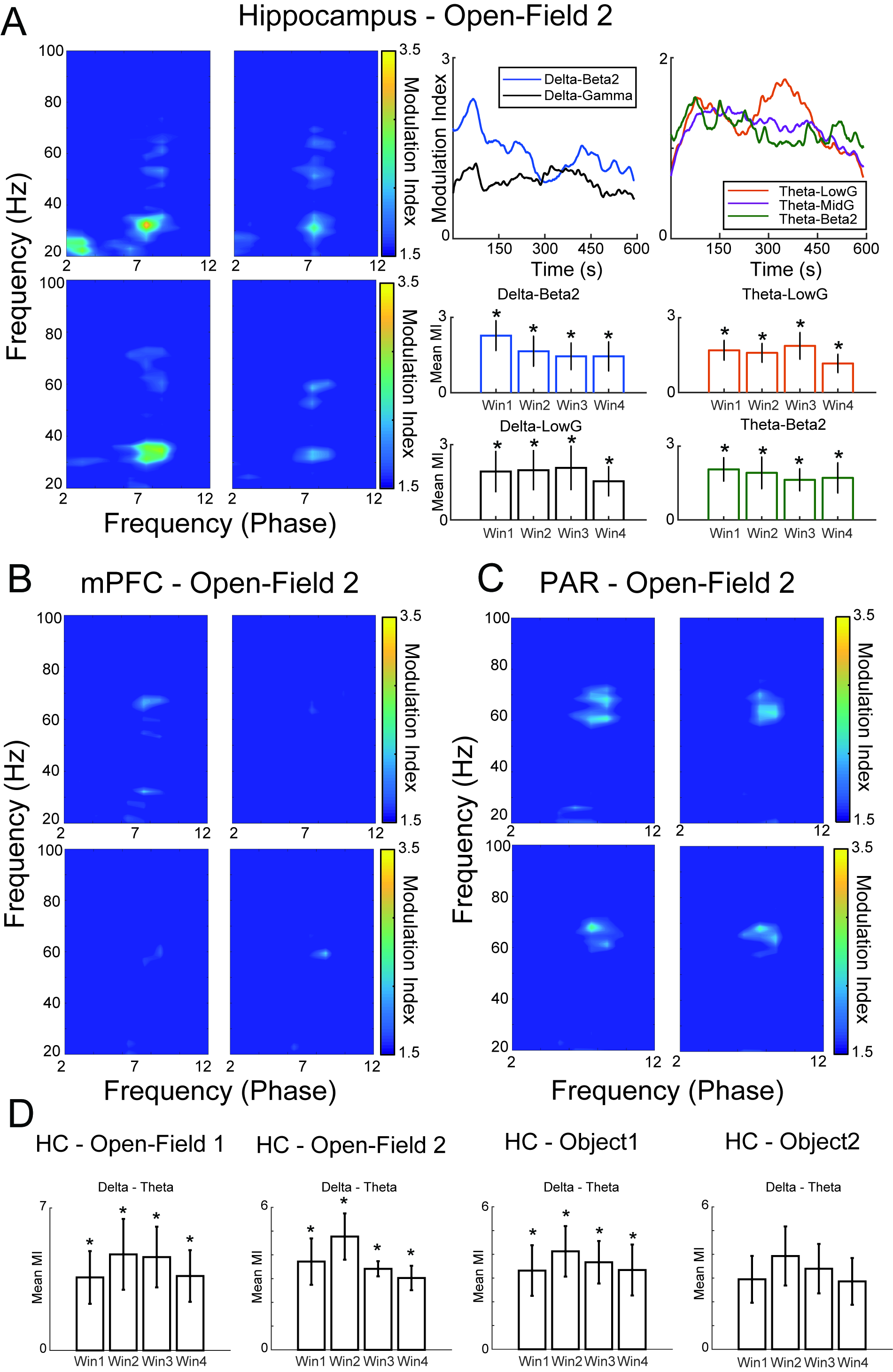

Supplement: Supplementary file 3 [file Image_3.TIF]
