# Supplementary material for: Beta2 Oscillations in Hippocampal-Cortical Circuits During Novelty Detection
Source: Front Syst Neurosci. 2021 Feb 16;15:617388. doi: 10.3389/fnsys.2021.617388 (PMC7921172; doi:10.3389/fnsys.2021.617388)
Supplement: Supplementary file 4 [file Table_1.PDF]

Table 1

**Exploration session**	Metric	ANOVA Statistics
**All Sessions**	**Delta Power** time window comparison	$F_{8.5}$ = 1.01, p = 0.43
**All Sessions**	**Theta Power** time window comparison	$F_{8.5}$ = 1.76, p = 0.12
**All Sessions**	**Beta1 Power** time window comparison	$F_{8.5}$ = 0.69, p = 0.67
**All Sessions**	**Beta2 Power** time window comparison	$F_{8.5}$ = 2.75, p = 0.02
**All Sessions**	**LowG Power** time window comparison	$F_{8.5}$ = 2.18, p = 0.06
**Open Field 1**	**Beta2 Power** time window comparison	$F_{3.8}$ = 4.14, p = 0.01
**Open Field 2**	**Beta2 Power** time window comparison	$F_{3.8}$ = 6.21, p = 0.003
**Object 1**	**Beta2 Power** time window comparison	$F_{3.6}$ = 3.79, p = 0.02;
**Object 2**	**Beta2 Power** time window comparison	$F_{3.6}$ = 3.03, p = 0.055

Table 1 – Table of statistics related to figure 1. Descriptive statistics and comparisons with different sessions spectral power values and beta2 power time windows. Black: Significant p values, Red: non-significant p values.
