# Supplementary material for: Beta2 Oscillations in Hippocampal-Cortical Circuits During Novelty Detection
Source: Front Syst Neurosci. 2021 Feb 16;15:617388. doi: 10.3389/fnsys.2021.617388 (PMC7921172; doi:10.3389/fnsys.2021.617388)
Supplement: Supplementary file 5 [file Table_2.PDF]

Table 2

Exploration session (Brain Region)	Metric	Time-Window ANOVA Statistics	Paired t-test against 0
**Open Field 1 (HC)**	**Delta/beta2** MI time window comparison	$F_{3,8}$ = 5.67, p = 0.004	Win1 - $t_{2,8}$=3.13, p= 0.018
			Win2 - $t_{2,8}$=3.56, p= 0.014
			Win3 - $t_{2,8}$=3.82, p= 0.014
			Win4 - $t_{2,8}$=2.91, p= 0.019
**Open Field 1 (HC)**	**Delta/lowG** MI time window comparison	$F_{3,8}$ = 3.69, p = 0.025	Win1 - $t_{2,8}$=3.72, p= 0.011
			Win2 - $t_{2,8}$=4.03, p= 0.011
			Win3 - $t_{2,8}$=3.08, p= 0.019
			Win4 - $t_{2,8}$=2.87, p= 0.020
**Open Field 1 (HC)**	**Theta/beta2** MI time window comparison	$F_{3,8}$ = 7.33, p = 0.001	Win1 - $t_{2,8}$=4.97, p= 0.004
			Win2 - $t_{2,8}$=4.17, p= 0.006
			Win3 - $t_{2,8}$=3.47, p= 0.011
			Win4 - $t_{2,8}$=1.74, p= 0.12
**Open Field 1 (HC)**	**Theta/lowG** MI time window comparison	$F_{3,8}$ = 8.51, p = 0.0004	Win1 - $t_{2,8}$=4.85, p= 0.002
			Win2 - $t_{2,8}$=4.47, p= 0.002
			Win3 - $t_{2,8}$=4.74, p= 0.002
			Win4 - $t_{2,8}$=3.80, p= 0.005
**Open Field 1 (HC)**	**Theta/midG** MI time window comparison	$F_{3,6}$ = 1.74, p = 0.18	Win1 - $t_{2,8}$=3.01, p= 0.020
			Win2 - $t_{2,8}$=2.96, p= 0.021
			Win3 - $t_{2,8}$=4.74, p= 0.021
			Win4 - $t_{2,8}$=2.93, p= 0.021
**Object 1 (HC)**	**Delta/beta2** MI time window comparison	$F_{3,6}$= 2.53, p = 0.08	Win1 - $t_{2,8}$=2.78, p= 0.027
			Win2 - $t_{2,8}$=2.62, p= 0.034
			Win3 - $t_{2,8}$=2.97, p= 0.027
			Win4 - $t_{2,8}$=3.28, p= 0.013
**Object 1 (HC)**	**Delta/lowG** MI time window comparison	$F_{3,6}$ = 1.44, p = 0.25	Win1 - $t_{2,8}$=6.98, p= 0.001
			Win2 - $t_{2,8}$=5.20, p= 0.005
			Win3 - $t_{2,8}$=3.31, p= 0.021
			Win4 - $t_{2,8}$=3.11, p= 0.017
**Object 1 (HC)**	**Theta/beta2** MI time window comparison	$F_{3,6}$= 3.83, p = 0.02	Win1 - $t_{2,8}$=3.75, p= 0.007
			Win2 - $t_{2,8}$=3.07, p= 0.017
			Win3 - $t_{2,8}$=2.28, p= 0.056
			Win4 - $t_{2,8}$=3.13, p= 0.016
**Object 1 (HC)**	**Theta/lowG** MI time window comparison	$F_{3,6}$= 4.13, p = 0.01;	Win1 - $t_{2,8}$=5.36, p= 0.001
			Win2 - $t_{2,8}$=4.24, p= 0.003
			Win3 - $t_{2,8}$=5.11, p= 0.001
			Win4 - $t_{2,8}$=3.61, p= 0.008
**Object 1 (HC)**	**Theta/midG** MI time window comparison	F3,6 = 0.40, p = 0.74	Win1 - $t_{2,8}$=4.03, p= 0.002
			Win2 - $t_{2,8}$=2.37, p= 0.030
			Win3 - $t_{2,8}$=2.51, p= 0.028
			Win4 - $t_{2,8}$=3.35, p= 0.008

Table 2 – Table of statistics related to figure 2. Descriptive statistics and comparisons with different MI time window analysis and the comparison with null hypothesis 0. Black: Significant p values, Red: non-significant p values.
