# Supplementary material for: Beta2 Oscillations in Hippocampal-Cortical Circuits During Novelty Detection
Source: Front Syst Neurosci. 2021 Feb 16;15:617388. doi: 10.3389/fnsys.2021.617388 (PMC7921172; doi:10.3389/fnsys.2021.617388)
Supplement: Supplementary file 6 [file Table_3.PDF]

Table 3

OpenField 1 Session			
Exploration session	**Metric**	**Time-Window ANOVA Statistics**	**Paired t-test against 0**
Open Field 1 mPFC	**Beta2 Power** time-window comparison	($F_{3,8}$ = 6.53, p = 0.002)	
Open Field 1 mPFC	**Delta/beta2** MI time window comparison	$F_{3,8}$ = 11.4, p = 0.00007	Win1 - $t_{2,8}$=4.09, p= 0.003
			Win2 - $t_{2,8}$=3.56, p= 0.026
			Win3 - $t_{2,8}$=3.82, p= 0.237
			Win4 - $t_{2,8}$=2.91, p= 0.501
Open Field 1 mPFC	**Delta/lowG** MI time window comparison	$F_{3,8}$ = 4.85, p = 0.0088	Win1 - $t_{2,8}$=2.71, p= 0.026
			Win2 - $t_{2,8}$=4.03, p= 0.033
			Win3 - $t_{2,8}$=3.08, p= 0.22
			Win4 - $t_{2,8}$=2.87, p= 0.67
Open Field 1 mPFC	**Theta/beta2** MI time window comparison	$F_{3,8}$ = 11.42, p > 0.0001	Win1 - $t_{2,8}$=3.81, p= 0.005
			Win2 - $t_{2,8}$=3.01, p= 0.016
			Win3 - $t_{2,8}$=1.03, p= 0.32
			Win4 - $t_{2,8}$=0.13, p= 0.89
Open Field 1 mPFC	**Theta/lowG** MI time window comparison	$F_{3,8}$ = 5.06, p = 0.007	Win1 - $t_{2,8}$=2.97, p= 0.017
			Win2 - $t_{2,8}$=2.98, p= 0.017
			Win3 - $t_{2,8}$=2.52, p= 0.035
			Win4 - $t_{2,8}$=1.75, p= 0.116
Open Field 1 mPFC	**Theta/midG** MI time window comparison	$F_{3,8}$ = 2.72, p = 0.066	Win1 - $t_{2,8}$=2.36, p= 0.045
			Win2 - $t_{2,8}$=2.48, p= 0.038
			Win3 - $t_{2,8}$=2.63, p= 0.030
			Win4 - $t_{2,8}$=2.88, p= 0.020
Open Field 1 PAR	**Beta2 Power** time-window comparison	($F_{3,8}$ = 1.07, p = 0.38)	
Open Field 1 PAR	**Delta/beta2** MI time window comparison	$F_{3,6}$= 14.62, p > 0.0001	Win1 - $t_{2,8}$=3.35, p= 0.006
			Win2 - $t_{2,8}$=2.65, p= 0.058
			Win3 - $t_{2,8}$=2.20, p= 0.213
			Win4 - $t_{2,8}$=1.36, p= 0.746
Open Field 1 PAR	**Delta/lowG** MI time window comparison	$F_{3,6}$ = 8.09, p = 0.0006	Win1 - $t_{2,8}$=2.67, p= 0.028
			Win2 - t2,8=1.80, p=0.108
			Win3 - $t_{2,8}$=3.31, p= 0.122
			Win4 - $t_{2,8}$=0.09, p= 0.929
Open Field 1 PAR	**Theta/beta2** MI time window comparison	$F_{3,6}$= 21.03, p > 0.0001	Win1 - $t_{2,8}$=5.15,p=0.0009
			Win2 - $t_{2,8}$=3.33, p= 0.010
			Win3 - $t_{2,8}$=1.77, p= 0.113
			Win4 - $t_{2,8}$=-0.20, p=0.845
Open Field 1		$F_{3,6}$= 8.56, p = 0.0004;	Win1 - $t_{2,8}$=3.12, p= 0.014

PAR	**Theta/lowG** MI time window comparison		Win2 - $t_{2,8}$=2.20, p= 0.058
			Win3 - $t_{2,8}$=1.80, p= 0.109
			Win4 - $t_{2,8}$=1.43, p= 0.189
Open Field 1 PAR	**Theta/midG** MI time window comparison	F3,6 = 3.98, p = 0.019	Win1 - $t_{2,8}$=3.26, p= 0.011
			Win2 - $t_{2,8}$=3.39, p= 0.009
			Win3 - $t_{2,8}$=3.49, p= 0.008
			Win4 - $t_{2,8}$=3.28, p= 0.011

Object 1 Session

Exploration session	**Metric**	**Time-Window ANOVA Statistics**	**Paired t-test against 0**
Object 1 mPFC	**Beta2 Power** time-window comparison	($F_{3,6}$ = 2.4, p = 0.10)	
Object 1 mPFC	**Delta/beta2** MI time window comparison	$F_{3,6}$ = 3.97, p = 0.021	Win1 - $t_{2,7}$=2.65, p= 0.040
			Win2 - $t_{2,7}$=2.14, p= 0.094
			Win3 - $t_{2,7}$=0.82, p= 0.253
			Win4 - $t_{2,7}$=2.13, p= 0.034
Object 1 mPFC	**Delta/lowG** MI time window comparison	$F_{3,6}$ = 0.99, p = 0.41	Win1 - $t_{2,7}$=2.18, p= 0.065
			Win2 - $t_{2,7}$=2.28, p= 0.056
			Win3 - $t_{2,7}$=2.14, p= 0.068
			Win4 - $t_{2,7}$=2.73, p= 0.029
Object 1 mPFC	**Theta/beta2** MI time window comparison	$F_{3,6}$ = 5.45, p > 0.006	Win1 - $t_{2,7}$=4.41, p= 0.003
			Win2 - $t_{2,7}$=2.70, p= 0.027
			Win3 - $t_{2,7}$=1.68, p= 0.144
			Win4 - $t_{2,7}$=2.13, p= 0.070
Object 1 mPFC	**Theta/lowG** MI time window comparison	$F_{3,6}$ = 1.21, p = 0.330	Win1 - $t_{2,8}$=2.97, p= 0.040
			Win2 - $t_{2,8}$=2.98, p= 0.046
			Win3 - $t_{2,8}$=2.52, p= 0.033
			Win4 - $t_{2,8}$=1.75, p= 0.032
Object 1 mPFC	**Theta/midG** MI time window comparison	$F_{3,6}$ = 1.45, p = 0.254	Win1 - $t_{2,8}$=2.58, p= 0.037
			Win2 - $t_{2,8}$=1.86, p= 0.104
			Win3 - $t_{2,8}$=2.05, p= 0.079
			Win4 - $t_{2,8}$=2.88, p= 0.021
Object 1 PAR	**Beta2 Power** time-window comparison	($F_{3,6}$ = 1.16, p = 0.35)	
Object 1 PAR	**Delta/beta2** MI time window comparison	$F_{3,6}$= 2.95, p = 0.060	Win1 - $t_{2,8}$=3.55, p= 0.012
			Win2 - $t_{2,8}$=2.65, p= 0.255
			Win3 - $t_{2,8}$=2.20, p= 0.323
			Win4 - $t_{2,8}$=1.36, p= 0.941
Object 1 PAR	**Delta/lowG** MI time window comparison	$F_{3,6}$ = 1.79, p = 0.18	Win1 - $t_{2,8}$=2.16, p= 0.073
			Win2 - $t_{2,8}$=1.80, p= 0.308
			Win3 - $t_{2,8}$=3.31, p= 0.106
			Win4 - $t_{2,8}$=0.09, p= 0.880

Object 1 PAR	**Theta/beta2** MI time window comparison	$F_{3,6}$= 0.57, p = 0.64	Win1 - $t_{2,8}$=2.26, p= 0.063
			Win2 - $t_{2,8}$=1.15, p= 0.290
			Win3 - $t_{2,8}$=2.80, p= 0.030
			Win4 - $t_{2,8}$=1.19, p= 0.278
Object 1 PAR	**Theta/lowG** MI time window comparison	$F_{3,6}$= 0.58, p = 0.63	Win1 - $t_{2,8}$=2.26, p= 0.173
			Win2 - $t_{2,8}$=1.15, p= 0.451
			Win3 - $t_{2,8}$=2.80, p= 0.079
			Win4 - $t_{2,8}$=1.19, p= 0.087
Object 1 PAR	**Theta/midG** MI time window comparison	$F_{3,6}$ = 1.37, p = 0.28	Win1 - $t_{2,8}$=3.26, p= 0.127
			Win2 - $t_{2,8}$=3.39, p= 0.221
			Win3 - $t_{2,8}$=3.49, p= 0.075
			Win4 - $t_{2,8}$=3.28, p= 0.100

Table 3 – Table of statistics related to figure 6. Descriptive statistics and comparisons with different MI time window analysis and the comparison with null hypothesis 0. Black: Significant p values, Red: non-significant p values.
