# Supplementary material for: Beta2 Oscillations in Hippocampal-Cortical Circuits During Novelty Detection
Source: Front Syst Neurosci. 2021 Feb 16;15:617388. doi: 10.3389/fnsys.2021.617388 (PMC7921172; doi:10.3389/fnsys.2021.617388)
Supplement: Supplementary file 7 [file Table_4.PDF]

Table 4

Exploration session	Metric	Time-Window ANOVA Statistics	Paired t-test against 0
**Open Field 2 (HC)**	**Delta/beta2** MI time window comparison	$F_{3,8}$ = 1.12, p = 0.359	Win1 - $t_{2,8}$=3.25, p= 0.011
			Win2 - $t_{2,8}$=2.74, p= 0.025
			Win3 - $t_{2,8}$=2.76, p= 0.024
			Win4 - $t_{2,8}$=2.82, p= 0.022
**Open Field 2 (HC)**	**Delta/lowG** MI time window comparison	$F_{3,8}$ = 0.22, p = 0.223	Win1 - $t_{2,8}$=2.85, p= 0.021
			Win2 - $t_{2,8}$=2.76, p= 0.024
			Win3 - $t_{2,8}$=2.59, p= 0.031
			Win4 - $t_{2,8}$=3.81, p= 0.005
**Open Field 2 (HC)**	**Theta/beta2** MI time window comparison	$F_{3,8}$ = 0.41, p = 0.74	Win1 - $t_{2,8}$=6.68, p> 0.001
			Win2 - $t_{2,8}$=3.45, p= 0.008
			Win3 - $t_{2,8}$=4.16, p= 0.003
			Win4 - $t_{2,8}$=3.04, p= 0.015
**Open Field 2 (HC)**	**Theta/lowG** MI time window comparison	$F_{3,8}$ = 2.44, p = 0.088	Win1 - $t_{2,8}$=4.77, p= 0.001
			Win2 - $t_{2,8}$=4.35, p= 0.002
			Win3 - $t_{2,8}$=4.32, p= 0.002
			Win4 - $t_{2,8}$=4.08, p= 0.003
**Open Field 2 (HC)**	**Theta/midG** MI time window comparison	$F_{3,8}$ = 1.44, p = 0.25	Win1 - $t_{2,8}$=2.93, p= 0.019
			Win2 - $t_{2,8}$=2.92, p= 0.019
			Win3 - $t_{2,8}$=2.96, p= 0.018
			Win4 - $t_{2,8}$=2.79, p= 0.023
**Open Field 2 (mPFC)**	**Delta/beta2** MI time window comparison	$F_{3,8}$= 0.86, p = 0.475	Win1 - $t_{2,8}$=1.83, p= 0.109
			Win2 - $t_{2,8}$=1.38, p= 0.208
			Win3 - $t_{2,8}$=2.40, p= 0.046
			Win4 - $t_{2,8}$=1.71, p= 0.129
**Open Field 2 (mPFC)**	**Delta/lowG** MI time window comparison	$F_{3,8}$ = 0.88, p = 0.464	Win1 - $t_{2,8}$=3.22, p= 0.042
			Win2 - $t_{2,8}$=1.88, p= 0.141
			Win3 - $t_{2,8}$=2.02, p= 0.174
			Win4 - $t_{2,8}$=1.44, p= 0.220
**Open Field 2 (mPFC)**	**Theta/beta2** MI time window comparison	$F_{3,8}$= 0.28, p = 0.83	Win1 - $t_{2,8}$=2.65, p= 0.032
			Win2 - $t_{2,8}$=2.52, p= 0.039
			Win3 - $t_{2,8}$=3.23, p= 0.014
			Win4 - $t_{2,8}$=2.50, p= 0.040
**Open Field 2 (mPFC)**	**Theta/lowG** MI time window comparison	$F_{3,8}$= 0.77, p = 0.510;	Win1 - $t_{2,8}$=3.78, p= 0.006
			Win2 - $t_{2,8}$=3.02, p= 0.019
			Win3 - $t_{2,8}$=2.21, p= 0.062
			Win4 - $t_{2,8}$=2.59, p= 0.035
**Open Field 2 (mPFC)**	**Theta/midG** MI time window comparison	$F_{3,8}$=0.36, p = 0.781	Win1 - $t_{2,8}$=4.03, p= 0.133
			Win2 - $t_{2,8}$=2.37, p= 0.061
			Win3 - $t_{2,8}$=2.51, p= 0.035
			Win4 - $t_{2,8}$=3.35, p= 0.063
**Open Field 2 (PAR)**	**Delta/beta2** MI time window comparison	$F_{3,8}$= 0.50, p = 0.683	Win1 - $t_{2,8}$=1.47, p= 0.191
			Win2 - $t_{2,8}$=1.57, p= 0.166

			Win3 - $t_{2,8}$=2.77, p= 0.032
			Win4 - $t_{2,8}$=2.46, p= 0.048
**Open Field 2 (PAR)**	**Delta/lowG** MI time window comparison	$F_{3,8}$ = 0.63, p = 0.599	Win1 - $t_{2,8}$=1.84, p= 0.114
			Win2 - $t_{2,8}$=0.48, p= 0.642
			Win3 - $t_{2,8}$=025, p= 0.810
			Win4 - $t_{2,8}$=1.39, p= 0.213
**Open Field 2 (PAR)**	**Theta/beta2** MI time window comparison	$F_{3,8}$= 0.39, p = 0.755	Win1 - $t_{2,8}$=2.62, p= 0.039
			Win2 - $t_{2,8}$=2.31, p= 0.059
			Win3 - $t_{2,8}$=2.98, p= 0.024
			Win4 - $t_{2,8}$=2.41, p= 0.052
**Open Field 2 (PAR)**	**Theta/lowG** MI time window comparison	$F_{3,8}$= 1.03, p = 0.40;	Win1 - $t_{2,8}$=1.44, p= 0.198
			Win2 - $t_{2,8}$=2.11, p= 0.078
			Win3 - $t_{2,8}$=1.60, p= 0.159
			Win4 - $t_{2,8}$=1.95, p= 0.098
**Open Field 2 (PAR)**	**Theta/midG** MI time window comparison	$F_{3,8}$=0.21, p = 0.881	Win1 - $t_{2,8}$=1.74, p= 0.132
			Win2 - $t_{2,8}$=2.10, p= 0.079
			Win3 - $t_{2,8}$=2.07, p= 0.083
			Win4 - $t_{2,8}$=2.14, p= 0.075
**Open Field 1 (HC)**	**Beta/Theta** MI time window comparison	$F_{3,8}$=1.41, p = 0.247	Win1 - $t_{2,8}$=2.89, p= 0.019
			Win2 - $t_{2,8}$=3.17, p= 0.013
			Win3 - $t_{2,8}$=3.71, p= 0.005
			Win4 - $t_{2,8}$=3.46, p= 0.008
**Open Field 2 (HC)**	**Beta/Theta** MI time window comparison	$F_{3,7}$=2.63, p = 0.081	Win1 - $t_{2,7}$=5.17, p= 0.002
			Win2 - $t_{2,7}$=8.19, p= 0.000
			Win3 - $t_{2,7}$=9.76, p= 0.000
			Win4 - $t_{2,7}$=5.76, p= 0.001
**Object 1 (HC)**	**Beta/Theta** MI time window comparison	$F_{3,7}$=0.63, p = 0.625	Win1 - $t_{2,7}$=5.21, p= 0.002
			Win2 - $t_{2,7}$=5.92, p= 0.001
			Win3 - $t_{2,7}$=4.65, p= 0.003
			Win4 - $t_{2,7}$=4.52, p= 0.004
**Object 2 (HC)**	**Beta/Theta** MI time window comparison	$F_{3,7}$=2.84, p = 0.073	Win1 - $t_{2,7}$=4.28, p= 0.008
			Win2 - $t_{2,7}$=4.07, p= 0.009
			Win3 - $t_{2,7}$=4.65, p= 0.005
			Win4 - $t_{2,7}$=3.92, p= 0.011

Table 4 – Table of statistics related to the supplementary figure 2. Descriptive statistics and comparisons with different MI time window analysis and the comparison with null hypothesis 0. Black: Significant p values, Red: non-significant p values.
